# Vaccination of koalas (*Phascolarctos cinereus*) against *Chlamydia pecorum* using synthetic peptides derived from the major outer membrane protein

**DOI:** 10.1371/journal.pone.0200112

**Published:** 2018-06-28

**Authors:** Sharon Nyari, Shahneaz Ali Khan, Galit Rawlinson, Courtney A. Waugh, Andrew Potter, Volker Gerdts, Peter Timms

**Affiliations:** 1 Faculty of Science, Health, Education and Engineering, University of the Sunshine Coast, Sippy Downs, Queensland, Australia; 2 Lone Pine Koala Sanctuary, Fig Tree Pocket, Queensland, Australia; 3 Vaccine and Infectious Disease Organisation–International Vaccine Centre (VIDO-InterVac), University of Saskatchewan, Saskatoon, Canada; Midwestern University, UNITED STATES

## Abstract

*Chlamydia pecorum* is a mucosal infection, which causes debilitating disease of the urinary tract, reproductive tract and ocular sites of koalas (*Phascolarctos cinereus*). While antibiotics are available for treatment, they are detrimental to the koalas’ gastrointestinal tract microflora leaving the implementation of a vaccine as an ideal option for the long-term management of koala populations. We have previously reported on the successes of an anti-chlamydial recombinant major outer membrane protein (rMOMP) vaccine however, recombinant protein based vaccines are not ideal candidates for scale up from the research level to small-medium production level for wider usage. Peptide based vaccines are a promising area for vaccine development, because peptides are stable, cost effective and easily produced. In this current study, we assessed, for the first time, the immune responses to a synthetic peptide based anti-chlamydial vaccine in koalas. Five healthy male koalas were vaccinated with two synthetic peptides derived from *C*. *pecorum* MOMP and another five healthy male koalas were vaccinated with full length recombinant *C*. *pecorum* MOMP (genotype G). Systemic (IgG) and mucosal (IgA) antibodies were quantified and pre-vaccination levels compared to post-vaccination levels (12 and 26 weeks). MOMP-peptide vaccinated koalas produced *Chlamydia*-specific IgG and IgA antibodies, which were able to recognise not only the genotype used in the vaccination, but also MOMPs from several other koala *C*. *pecorum* genotypes. In addition, IgA antibodies induced at the ocular site not only recognised recombinant MOMP protein but also, whole native chlamydial elementary bodies. Interestingly, some MOMP-peptide vaccinated koalas showed a stronger and more sustained vaccine-induced mucosal IgA antibody response than observed in MOMP-protein vaccinated koalas. These results demonstrate that a synthetic MOMP peptide based vaccine is capable of inducing a *Chlamydia-*specific antibody response in koalas and is a promising candidate for future vaccine development.

## Introduction

*Chlamydia* (*C*) continues to be one of the major factors threatening the long-term survival of the koala (*Phascolarctos cinereus*). *C*. *pecorum* is primarily considered to be a sexually transmitted infection however, congenital transmission has also been shown [1]. *C*. *pecorum* is a mucosal infection, which causes debilitating disease at the urinary tract, reproductive tract and ocular sites of koalas [2]. When left untreated, *C*. *pecorum* infection can lead to cystitis, infertility and blindness [2–5]. The current treatment for *Chlamydia* in koalas involves the use of antibiotics, which can be detrimental to the koalas’ gastrointestinal tract microflora, which is essential for the digestion of their diet of eucalyptus leaves [6–8]. Although antibiotics can be a useful treatment, they offer no long-term protection from subsequent infections and have limited effect on severe cases of chlamydiosis [9]. Furthermore, as *Chlamydia* can be asymptomatic, showing no overt signs of disease in up to 50% of infected koalas, many infected koalas go untreated [1].

Our group has been working towards the successful development of an anti-chlamydial vaccine with considerable progress [10–20]. Until now, the vaccine has been composed of recombinant proteins derived from the full length chlamydial major outer membrane protein (MOMP) [21]. Previous vaccine trials have shown that, a) the vaccine is safe to use, in both healthy and infected koalas [11], b) there is a level of cross protection against other koala-*C*. *pecorum* genotypes [12], c) both humoral and cellular immune responses are stimulated [14, 15], d) the vaccine-induced immune responses are long lasting [14], and e) vaccination has both prophylactic as well as therapeutic effects [19]. Although the current recombinant based vaccine has shown considerable promise, its production is at a research level and scale up for wider use will be challenging. One alternative would be the development of a synthetic, peptide based vaccine. Peptide vaccines are becoming increasingly popular, primarily because peptide based antigens are, a) easier to produce, b) cost effective, c) customised, d) typically water-soluble, e) stable, and f) able to be freeze dried for long-term storage [22]. Furthermore, it has also been suggested that dendritic cells (DC) can process synthetic peptides more efficiently than full length proteins [23]. This would provide an ideal advantage as antigen presenting cells (APCs) are responsible for initiating adaptive T-cell responses, which are thought to be needed for cellular and/or humoral immunity to *Chlamydia* [22, 24]. However, peptide based antigens are often recognised as being poor immunogens, lacking essential immunostimulatory properties required for effective immune stimulation [22, 25–28]. The addition of an appropriate delivery system or adjuvant is therefore essential. Whilst there are a number of adjuvants suitable for use in animals the choice of adjuvant for use in humans is limited due to their adverse side effects and toxicity [29, 30]. For this reason, careful consideration should be taken when selecting an appropriate adjuvant. Peptide vaccines have also been shown to induce tolerance and autoimmunity, post vaccination [30–34]. This is of particular importance when developing anti-cancer peptide-based vaccines as tumor-associated antigens can also be expressed in normal cells where self-recognition can lead to tolerance or an induced immune response could lead to autoimmunity [30].

Developing a successful peptide based vaccine requires the identification of key epitopes responsible for producing strong humoral and cell mediated immunity against *C*. *pecorum*. Our progression towards a peptide based vaccine, has identified the location of B and T cell epitopes within the full length MOMP from a diverse range of *C*. *pecorum* genotypes, with a focus on the immunostimulatory epitopes located within both the variable and conserved regions of MOMP [13, 35]. Further analysis was then performed to identify which epitopes were responsible for inducing antibodies with neutralising capabilities, essential for an effective vaccine. This led to the identification of a unique set of epitopes, all contained within the conserved region of MOMP, that induced antibodies capable of neutralising whole *C*. *pecorum* elementary bodies (EBs) [15, 16]. As *C*. *pecorum* vaccinated koalas have previously been shown to cross-recognise other *C*. *pecorum* genotypes [12] and given this unique set of neutralising epitopes that reside within the conserved region of MOMP, it is equally expected that a peptide vaccine, based on these epitopes, would also show cross-protective abilities against other *C*. *pecorum* genotypes. In this study, we present for the first time, the humoral responses in koalas vaccinated with a synthetic peptide antigen, derived from the conserved region of whole *C*. *pecorum* MOMP, combined with a Tri-Adjuvant [36]. We have shown that our peptide antigen is capable of inducing a systemic IgG antibody response as well as a mucosal IgA antibody response at the ocular site.

## Materials and methods

### Animals

Ten healthy captive adult male koalas were used for this study. All koalas had been bred and housed at, Lone Pine Koala Sanctuary (LPKS), Fig Tree Pocket, Brisbane, Queensland, Australia. A full veterinary health check was performed on all koalas before being given approval to participate in the vaccine trial. Enclosures and koala husbandry followed the Code of Practice for wildlife care (Queensland). Koala enclosures consisted of a sand or concrete floor with either wooden, brick, colourbond or glass walls with a tin or sail roof. Koalas remained housed in their usual enclosure with other koalas, which all met or exceeded zoo standards for koala exhibit size. They were supplied with fresh eucalyptus leaves daily, with the water in the leaf holding pots and water dishes topped up two to three times a day. The floors were cleaned once a day, and the koala climbing poles were cleaned once a week with disinfectant. All koalas were individually checked daily by a senior koala keeper and cared for by their regular koala keepers. All procedures relating to this study were approved by the University of the Sunshine Coast (USC) Animal Ethics Committee (Animal Ethics permit number AN/S/15/42) and by the Queensland Government (Scientific Purposes Permit number WISP16718315).

### *Chlamydia pecorum* MOMP purification

The protein antigen vaccine consisted of recombinant MOMP G. ELISA’s were performed using recombinant MOMP genotypes, A, F and G. *Chlamydia pecorum* MOMP proteins, genotype A, F and G, was purified as previously described by Kollipara *et al*. (2012) [11].

### Peptide synthesis and alignment

**Fig 1:**
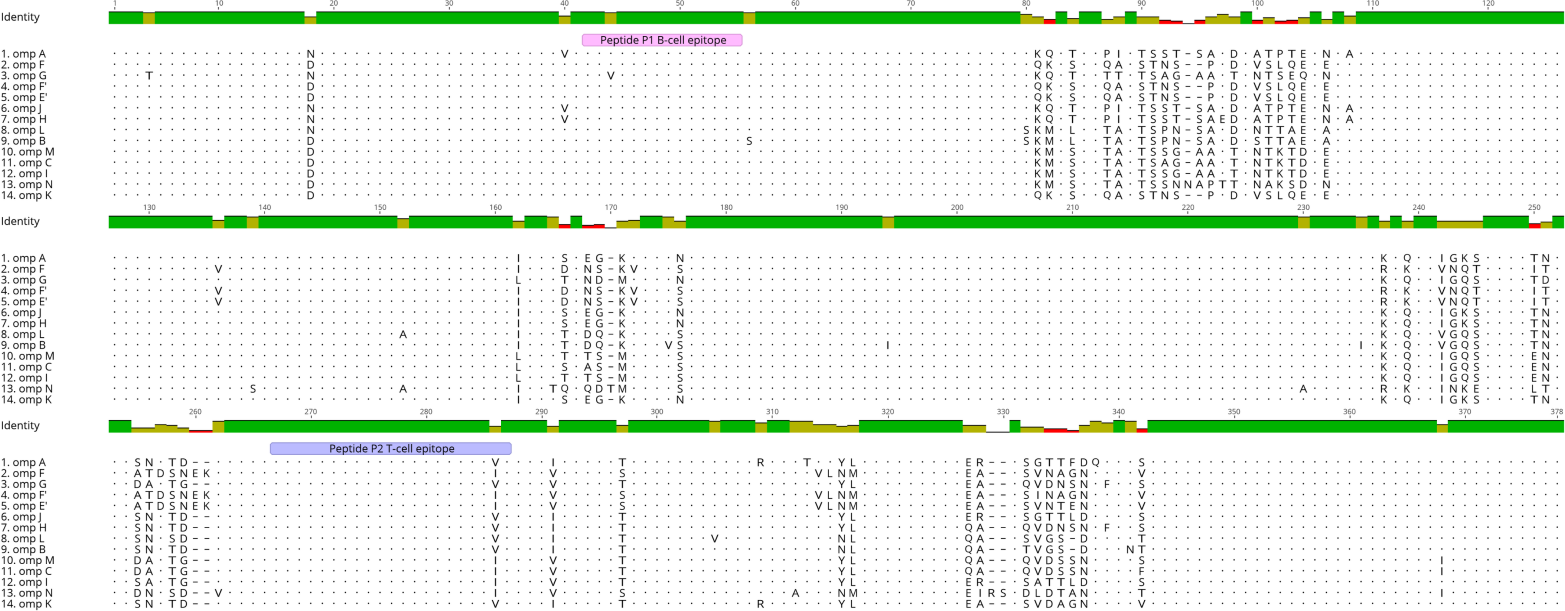
Vaccine peptide antigens are located within the conserved regions of the chlamydial MOMP. Sequence alignment showing the location of the vaccine peptide antigens at 45–55 (P1) and 265–285 (P2) are both within the conserved regions of the major outer membrane protein across the 14 koala *Chlamydia pecorum* ompA genotypes (A, F, G, F’, E’, J, H, L, B, M, C, I, N and K).

The peptide antigen vaccine consisted of two synthetic peptides located within the conserved regions of MOMP. P1, a 14 amino acid sequence, H-EGMSGDPCDPCATW-OH and P2, a 21 amino acid sequence H-INYHEWQVGAALSYRLNMLIP-OH (Fig 1). For use in the ELISA assays, both P1 and P2 were constructed as Biotin molecules linked to a serine glycine (SGSG) spacer. Peptides were reconstituted using endotoxin-free water. All peptides were produced by Mimotopes (Melbourne, Australia).

Koala *C*. *pecorum* ompA genotypes were compared for similarity within the conserved regions, where the two synthetic peptides (P1 and P2) are located. In total, 14 *C*. *pecorum* ompA genotypes (A, F, G, F’, E’, J, H, L, B, M, C, I, N and K), were retrieved from GenBank for sequence comparison (Fig 1). All *C*. *pecorum* ompA sequences can be found through GenBank using accession numbers, AGV52054 (A), AGV52057 (F), AGV52059 (G), AGV52058 (F’), AGV52056 (E’), AGV52062 (J), AGV52060 (H), AMO26059 (L), AGV52055 (B), AMO26056 (M), AMO26054 (C), AGV52061 (I), AMO26053 (N) and AGV52063 (K). Sequence alignment was performed using Geneious 9.1 software.

### Immunisation schedule and sample collection

Koalas were randomly assigned to two groups of five animals. One group (n = 5) received a single subcutaneous injection containing recombinant MOMP G (50μg) with Tri-Adjuvant (MOMP-protein vaccinated group), and the other group (n = 5) received a single subcutaneous injection containing synthetic peptides P1 and P2 (50μg or each peptide) (MOMP-peptide vaccinated group) combined with Tri-Adjuvant and made up to a total volume of 500μL with sterile endotoxin-free PBS. The Tri-Adjuvant contained a 1:2:1 ratio of Poly I;C (250μg), Host Defence Peptide–Innate Defence Regulator IDR-1002 (500μg), and Polyphosphazene EP3 (250μg) all produced and supplied by VIDO-Intervac (University of Saskatchewan, Saskatoon, SK, CA). Each vaccine was prepared in a 2mL sterile endotoxin free amber glass vials, stored on ice and administered within 2 hours of preparation.

All koalas were sampled pre-vaccination and again at 12 and 26 weeks post-vaccination. Samples were collected from the koalas whilst they were being restrained by an experienced staff member, as per standard Lone Pine Koala Sanctuary procedure. Briefly, koalas were held facing outwards from the handler and seated on a table. Forelimb and hindlimb were held together, right with right and left with left in the handlers’ respective right and left hands. Antiseptic was applied to the forelimb and whole blood (3mL) was collected from either the left or right cephalic vein. Whole blood was then placed into EDTA collection tubes (Interpath Services) and stored at 4°C until centrifugation where the plasma was removed and then stored at -20°C. Swabs were collected from the ocular site (Aluminium rayon swabs; Copan), placed into 1% protease inhibitor cocktail and stored at -20°C until processed.

### Koala specific *Chlamydia pecorum* IgG ELISA

The IgG ELISA assay was performed to determine the systemic antibody response utilising recombinant MOMP proteins A, F and G, from plasma samples, collected pre-vaccination and again at 12 and 26 weeks post-vaccination. Initially, 96 well plates (Greiner Bio-One medium binding) where coated with 50μL of carbonate-bicarbonate coating buffer containing, 2μg/well of recombinant MOMP G, then incubated at 4°C overnight. After incubation, wells were emptied then coated with 100μL per well of blocking buffer consisting of 5% skim milk in PBS containing 0.01% Tween-20 then incubated for 2 hours at 37°C. After incubation, wells were emptied then 1:3 serially diluted plasma, with dilutions starting at 1:50, was added in duplicate then incubated for 1 hour at 37°C. After incubation, wells were washed 3 times with PBS containing 0.05% Tween-20 then coated with 50μL/well of sheep anti-koala IgG diluted 1:8000 in PBS containing 0.01% Tween-20 then incubated for 1 hour at 37°C. After incubation, wells were washed 3 times with PBS containing 0.05% Tween-20 then coated with 50μL/well of HRP-conjugated donkey anti-sheep IgG diluted 1:20000 (Abcam) in PBS containing 0.01% Tween-20 then incubated for 1 hour at 37°C. After incubation, wells were washed 3 times with PBS then 50μL/well of TMB substrate (Sigma-Aldrich) was added and incubated at room temperature for 30 mins before stopping the reaction with 50μL/well of 1M sulphuric acid. The end point titre (EPT) was calculated as per [20].

### Koala specific *Chlamydia pecorum* IgA ELISA

The IgA ELISA assay was performed to determine the mucosal antibody response utilising recombinant MOMP protein, and heat inactivated semi-purified *C*. *pecorum* G EBs (purified as per Carey *et al*. (2010)) [10] on ocular swab samples, stored in 1% PIC, collected at pre-vaccination and again at 12 and 26 weeks post-vaccination. Initially, 96 well plates (Greiner Bio-One medium binding) where coated with 50μL of carbonate-bicarbonate coating buffer containing either, 2μg/well of recombinant MOMP G or 50000 IFU/well of heat inactivated semi-purified *C*. *pecorum* G EBs, then incubated at 4˚C overnight. After incubation, wells were emptied then coated with 100μL per well of blocking buffer consisting of 5% skim milk in PBS containing 0.01% Tween-20 then incubated for 2 hours at 37˚C. After incubation, wells were emptied then 50μL/well of swab sample solution, defrosted at room temperature then vortexed for 3 minutes, was added in duplicate then incubated for 1 hour at 37°C. After incubation, wells were washed 3 times with PBS containing 0.05% Tween-20 then coated with 50μL/well of rabbit anti-koala IgA diluted 1:3000 in PBS containing 0.01% Tween-20 then incubated for 1 hour at 37°C. After incubation, wells were washed 3 times with PBS containing 0.05% Tween-20 then coated with 50μL/well of HRP-conjugated goat anti-rabbit IgG (ab6721; Abcam) diluted 1:20000 in PBS containing 0.01% Tween-20 then incubated for 1 hour at 37°C. After incubation, wells were washed 3 times with PBS then 50μL/well of TMB substrate (Sigma-Aldrich) was added and incubated at room temperature for 30 mins before stopping the reaction with 50μL/well of 1M sulphuric acid. The optical density (OD) was measured at 450nm and the absorbance value was calculated as the mean of duplicate samples minus the mean of the no sample control wells.

### Koala specific synthetic peptide IgG and IgA ELISA

Peptide ELISA’s were performed to determine the antibody response to P1 and P2, as described by Bommana *et al*. (2017) [37] with the following modifications. Streptavidin plates were initially coated with either, 1.5μg/well of P1 or 1.5μg/well of P2. For the IgG ELISA, 100μL/well of 1:3 serially diluted plasma, starting with a 1:50 dilution, was added in duplicate, following incubation, 100μL/well of sheep anti-koala IgG, diluted 1:8000, was added followed by a final incubation of 100μL/well of HRP-conjugated donkey anti-sheep IgG diluted 1:20000 (Abcam). For the IgA ELISA, 50μL/well of swab sample solution, defrosted at room temperature then vortexed for 3 minutes, was added in duplicate, following incubation, 100μL/well rabbit anti-koala IgA, diluted 1:3000, was added followed by a final incubation of 100μL/well HRP-conjugated goat anti-rabbit IgG (ab6721; Abcam) diluted 1:20000. The optical density (OD) was measured at 450nm and the absorbance value was calculated as the mean of duplicate samples minus the mean of the no sample control wells.

### Statistical analysis

All statistical analysis was performed using GraphPad Prism version 7 (GraphPad Software, LaJolla, CA, USA). To evaluate the difference between time-points of each group, a one-way ANOVA Tukey’s multiple comparison test was performed with P-values set at *p<0.05, **p<0.01, ***p<0.005.

## Results

### Systemic IgG antibody response of vaccinated koalas to full length recombinant MOMP protein

**Fig 2:**
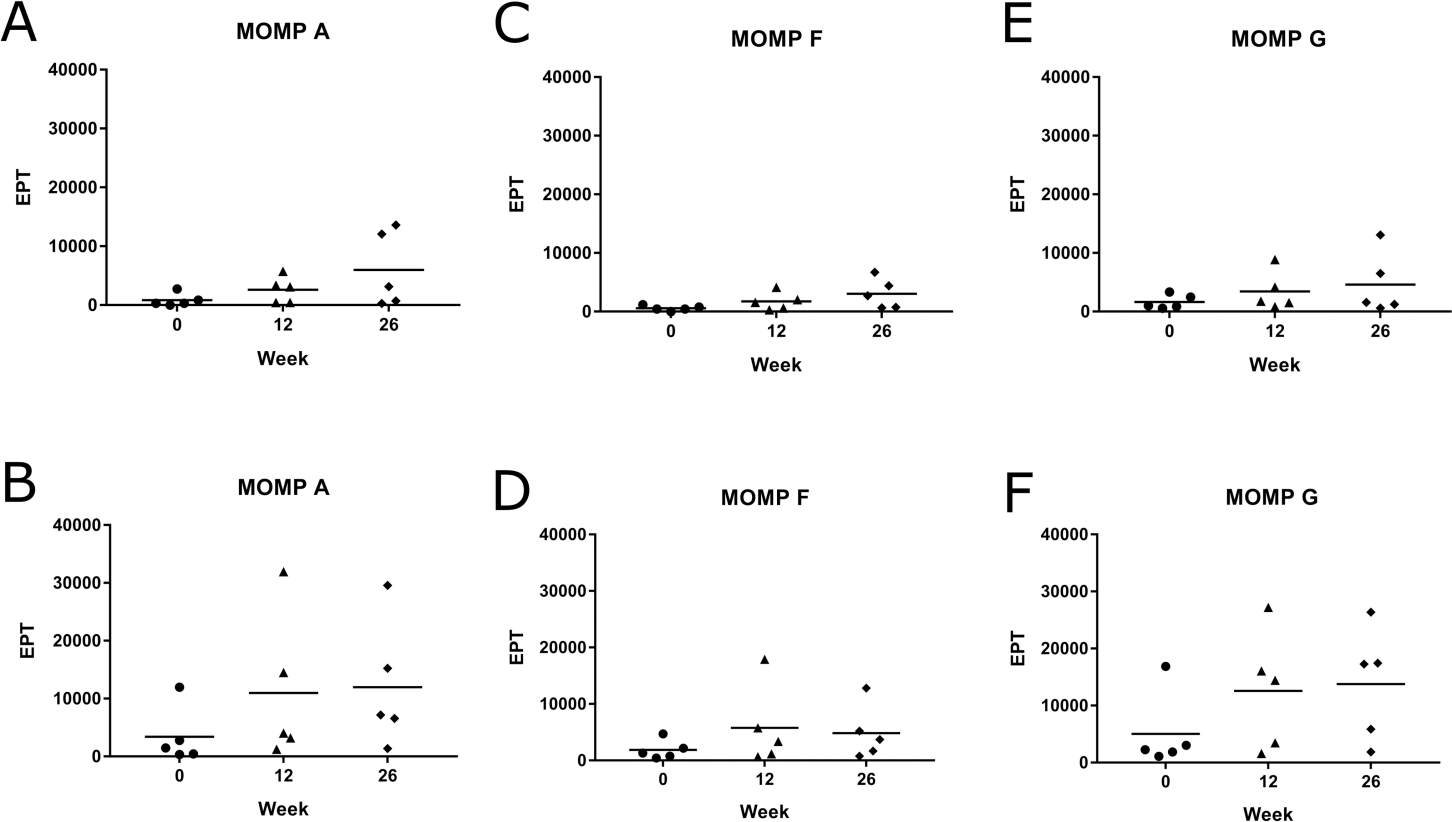
MOMP-peptide vaccinated koalas (*Phascolarctos cinereus*) produced a systemic IgG antibody response to rMOMP proteins. Systemic IgG antibody response (from plasma) against recombinant protein from *Chlamydia pecorum* MOMP genotypes A, F and G measured pre-vaccination and at 12 and 26 weeks post-vaccination. Samples were analysed by ELISA and measurments are shown as end point titre (EPT). (A, C and E) MOMP-peptide vaccinated koalas (n = 5) response against recombinant MOMP genotypes A, F and G, respectively. (B, D and F) MOMP-protein vaccinated koalas (n = 5) response against recombinant MOMP genotypes A, F and G, respectively.

We initially assessed the ability of vaccine induced antibodies to recognise full length recombinant MOMP protein in an ELISA. The systemic IgG antibody response was measured against recombinant MOMP protein from three different *C*. *pecorum* genotypes (A, F and G) to assess their ability to cross-recognise varying MOMP genotypes (Fig 2). These genotypes were chosen as representatives of the previously identified genotypes circulating in wild koala populations, particularly in northern Australia. MOMP-peptide vaccinated koalas produced a good systemic IgG response, to all three recombinant MOMP genotypes (A, F and G) (Fig 2A, 2C and 2E), with the response increasing gradually to 26 weeks post-vaccination. As expected, MOMP-protein vaccinated koalas also produced a strong systemic IgG response, to all three recombinant MOMP genotypes (A, F and G) (Fig 2B, 2D and 2F), which was, overall, slightly stronger than that produced in the MOMP-peptide vaccinated group. Eventhough the vaccine was designed against the *C*. *pecorum* genotype G MOMP sequence, both groups produced antibodies that also recognised MOMP protein from *C*. *pecorum* genotypes A and F, in addition to G.

### Systemic IgG antibody response of vaccinated koalas to the synthetic peptides P1 and P2

**Fig 3:**
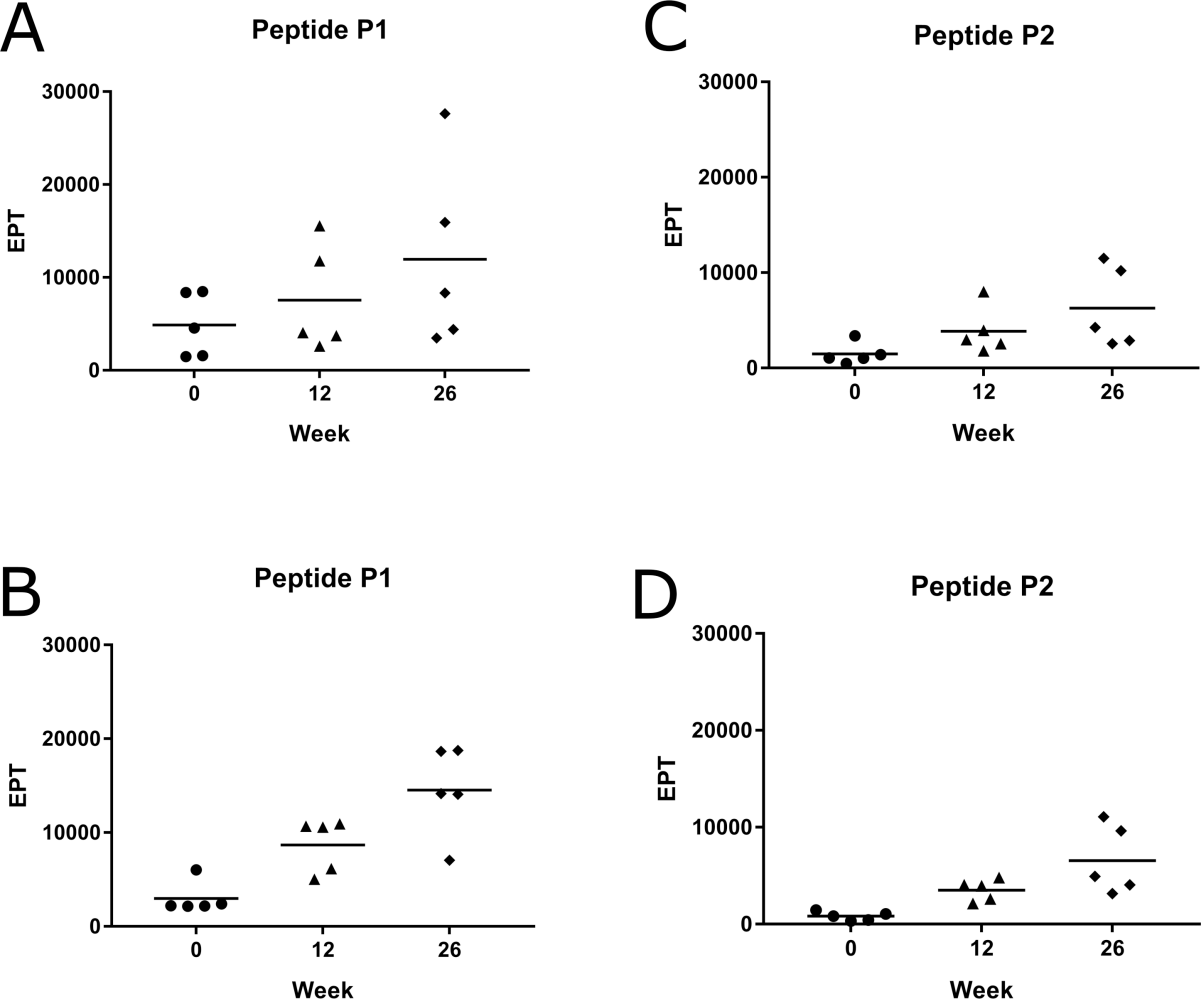
MOMP-peptide vaccinated koalas (*Phascolarctos cinereus*) produced a systemic IgG antibody response to the peptides P1 and P2. Systemic IgG antibody response (from plasma) against two different synthetic peptides (P1 and P2) measured pre-vaccination and at 12 and 26 weeks post-vaccination. Samples were analysed by ELISA and measurments are shown as EPT. (A) MOMP-peptide vaccinated koalas (n = 5) response against P1. (B) MOMP-protein vaccinated koalas (n = 5) response against P1. (C) MOMP-peptide vaccinated koalas (n = 5) response against P2. (D) MOMP-protein vaccinated koalas (n = 5) response against P2.

We then determined the level of antibodies produced post-vaccination that specifically recognised the peptides P1 and P2. All MOMP-peptide vaccinated koalas made an increasing vaccine-induced systemic IgG antibody response, from pre-vaccination to 26 weeks post-vaccination, when tested against each peptide (P1 and P2) (Fig 3A and 3C). The MOMP-protein vaccinated koalas also produced vaccine-induced systemic IgG antibodies, which recognised the peptides, P1 and P2 (Fig 3B and 3D) that increased to 26 weeks post-vaccination. Intersestingly, similar results were seen between the two vaccinated groups from pre-vaccination to 26 weeks post-vaccination.

### Mucosal IgA antibody response in vaccinated koalas to full length recombinant MOMP protein, synthetic peptides P1 and P2 and whole *Chlamydia pecorum* elementary bodies

**Fig 4:**
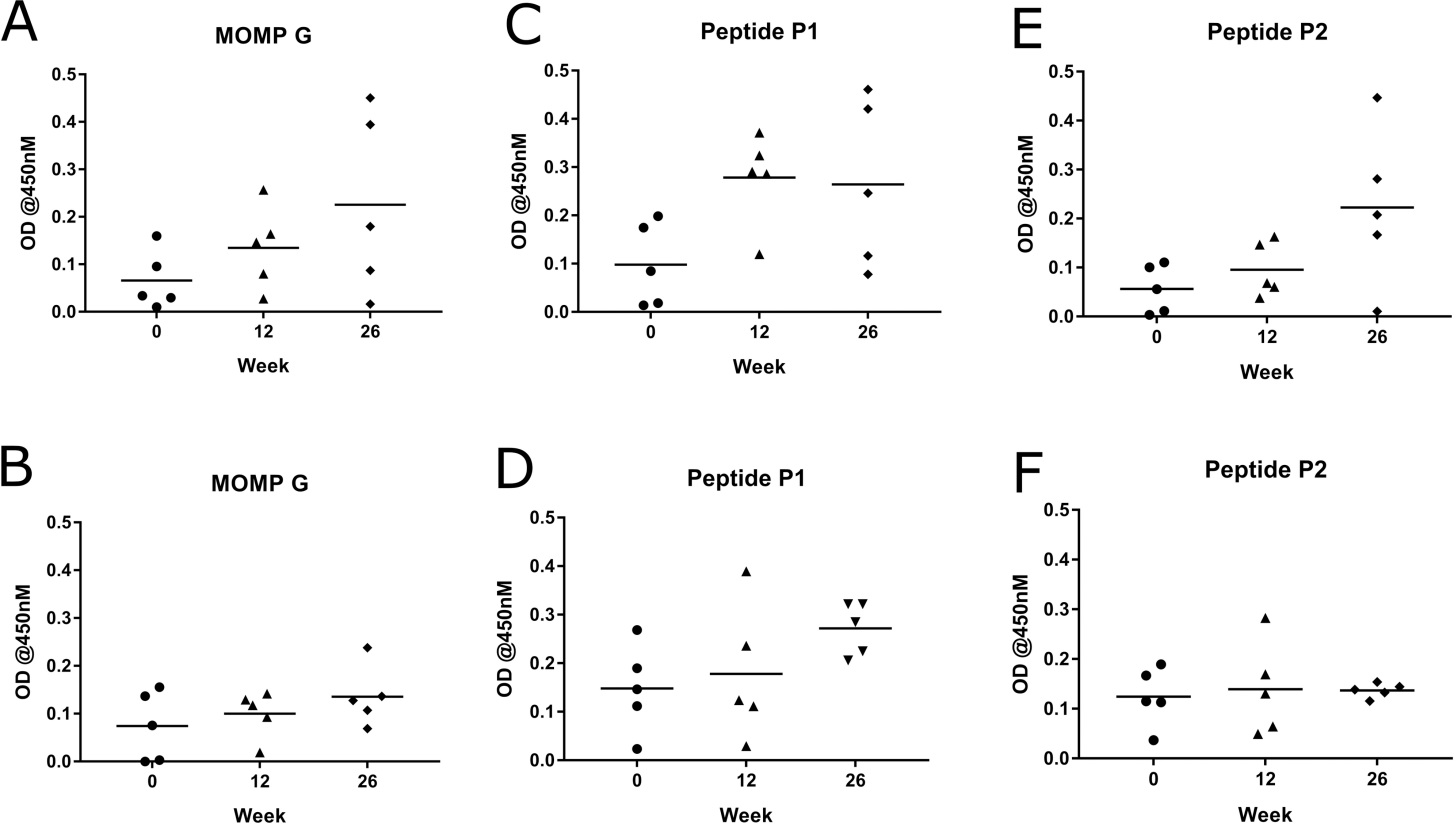
MOMP-peptide vaccinated koalas (*Phascolarctos cinereus*) produced a stronger mucosal IgA antibody response to rMOMP protein than MOMP-protein vaccinated koalas (*Phascolarctos cinereus*). Mucosal IgA antibody response to recombinant MOMP (G) and synthetic peptides (P1 and P2) in ocular swab samples collected pre-vaccination and 12 and 26 weeks post-vaccination. Samples were analysed by ELISA and are shown as optical density (OD) measured at 450nM. (A, C and E) MOMP-peptide vaccinated koalas (n = 5) response to recombinant MOMP G, peptide P1 and peptide P2, respectively. (B, D and F) MOMP-protein vaccinated koalas (n = 5) response to recombinant MOMP G, peptide P1 and peptide P2, respectively.

**Fig 5:**
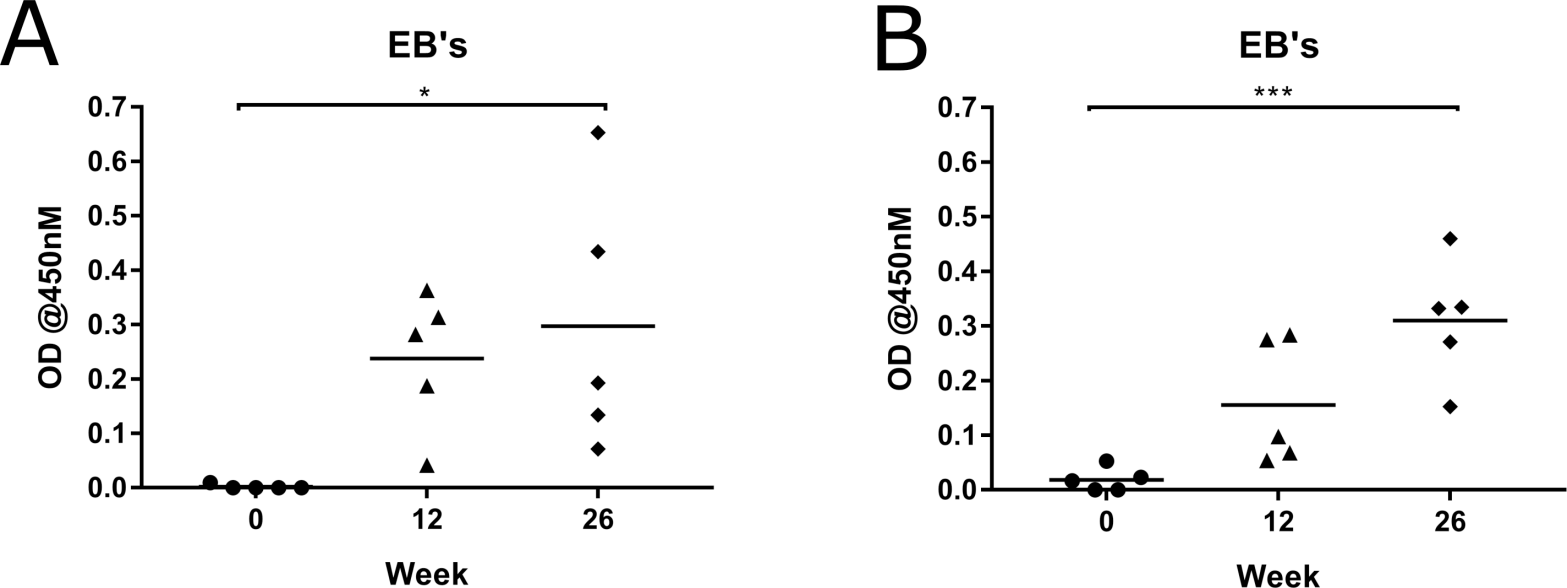
MOMP-peptide vaccinated koalas (*Phascolarctos cinereus*) produced a stronger mucosal IgA antibody response to whole *Chlamydia pecorum* elementary bodies than MOMP-protein vaccinated koalas (*Phascolarctos cinereus*). Mucosal IgA antibody response to heat inactivated *Chlamydia pecorum* elementary bodies from genotype G in ocular swab samples collected pre-vaccination and 12 and 26 weeks post-vaccination. Samples were analysed by ELISA and are shown as OD measured at 450nM. (A) MOMP-peptide vaccinated koalas (n = 5) response with a P value 0.0009. (B) MOMP protein-vaccinated koalas (n = 5) response with a P value 0.0299.

Because *Chlamydia* is a mucosal pathogen, causing disease at the ocular and urogenital sites, it is important for any vaccine to induce the correct immune response at these mucosal sites. We analysed the IgA antibody response to vaccination at the ocular site in this study. The mucosal IgA antibody response was measured against *C*. *pecorum* recombinant MOMP protein (G), the two synthetic peptides (P1 and P2) and whole *C*. *pecorum* EBs (G). The MOMP-peptide vaccinated koalas produced *Chlamydia*-specific mucosal IgA antibodies at the ocular site to all three forms of chlamydial antigen tested. They produced antibodies that recognised full length recombinant MOMP protein as well as peptides P1 and P2 (Fig 4A, 4C and 4D). Importantly, they produced a significant increase in IgA antibodies that recongised whole native chlamydial EBs (P = 0.0299) (Fig 5A). Perhaps not surprisingly, the MOMP-protein vaccinated koalas also produced IgA antibodies at the ocular site to *C*. *pecorum* recombinant MOMP protein (G), two synthetic peptides (P1 and P2) (Fig 4B, 4D and 4F) and a significant increase to whole native chlamydial EBs (P = 0.0009) (Fig 5B). Interestingly, although some koalas showed a stronger vaccine-induced mucosal IgA antibody response than others, at 12 and 26 weeks post-vaccination, some of the MOMP-peptide vaccinated koalas made a stronger and more sustained antibody response, compared to the MOMP-protein vaccinated group.

## Discussion

Wild koala populations continue to have significant levels of infection with *C*. *pecorum* and as a result are suffering debilitating disease, which is threatening their long-term survival [1, 38, 39]. In many populations, these levels of infection and disease are actually higher than previously reported [1] and current treatment options are showing little to no impact on the decline in the level of infection and disease, with hospital admission records remaining stable over time [39]. The widespread implementation of a vaccine in wild koalas could offer the protection needed to reverse this progression. To date, previous rMOMP protein vaccine trials conducted on both infected and diseased wild koala populations have been very successful [11, 13, 15, 16, 18, 19]. These trials have shown that a rMOMP protein vaccine stimulates the immune system and is responsible for an increase in neutralising antibodies [11, 15, 18]. Vaccinated koalas have shown a decrease in their chlamydial infectious load [18, 19] as well as ocular disease status [19], post-vaccination, and importantly, rMOMP vaccinated wild koalas have also shown a decrease in the progression to disease over a 12 month period [18–20]. However, in spite of this success, there are many challenges in producing and implementing a recombinant protein format vaccine on a wider scale. A synthetic peptide based vaccine could overcome some of these challenges, assuming that it induces a strong and relevant immune response [22]. The development of a peptide based anti-chlamydial vaccine that can elicit the same, or even stronger immune responses as the current *C*. *pecorum* MOMP vaccine, with the potential to be mass produced, would be an ideal candidate for future anti-chlamydial vaccine development. In this study, we have shown that a vaccine consisting of two relatively short peptides, derived from the full length MOMP, is capable of inducing an immune response in koalas up to 26 weeks post-vaccination. We have shown a mucosal IgA antibody response to full length rMOMP (G), in both the MOMP-peptide and MOMP-protein vaccinated koalas. Most importantly, for the first time, we found that the MOMP-peptide vaccinated koalas produced a mucosal IgA antibody response to whole chlamydial EBs with some MOMP-peptide vaccinated koalas showing a stronger response than the MOMP-protein vaccinated koalas. Furthermore, our study has also shown that the MOMP derived peptide vaccine is safe to use in koalas with no adverse effects reported in any of the koalas involved in this trial.

For the successful development of an anti-*C*. *pecorum* vaccine, it is important that the vaccine antigen is specific, induces a strong humoral and cell mediated response and can elicit long-lasting immunity. Additionally, it is advantageous that an anti-chlamydial vaccine antigen is capable of cross recognising other *C*. *pecorum* strains circulating in wild koala populations causing infection and disease [1, 38]. Previously, we have shown that using a full length rMOMP protein induced antibodies that could recognise other chlamydial genotypes (A, F and G) [12]. This current study also found that using just two peptides (14 aa and 21 aa in length) located at positions 42–55 (P1) and 265–285 (P2) in the MOMP, can also induce antibodies to epitopes of chlamydial genotypes A, F and G. Presumably, this is because these peptides were designed from regions of MOMP that are conserved across genotypes A, F and G. By carefully selecting peptides located in these conserved regions of MOMP, we further predict that these peptides should also be able to cross-recognise other *C*. *pecorum* genotypes, as these regions are also conserved across all 14 currently known koala *C*. *pecorum* ompA genotypes.

As *C*. *pecorum* is a mucosal infection, it is essential that an anti-chlamydial vaccine elicits a strong mucosal IgA antibody response. Secretory IgA found in mucosal surfaces is the first line of defence against invading pathogens and works to prevent infection by blocking the attachment of pathogens to epithelial cells and by eliminating pathogens from the mucosal surface [40, 41]. Studies in mice have shown that mucosal IgA was responsible for protective immunity when challenged [42] and in another study, secretory IgA was shown to have an effect on reducing the chlamydial infectious load [43]. Furthermore, a recent study conducted by Desclozeaux *et al*. (2017) [20] also showed that IgA could possibly play a role in lowering the chlamydial burden in koalas. In our current study, we have shown a strong mucosal IgA antibody response by vaccinated koalas that recognises rMOMP (G) protein and peptide (P1 and P2) antigens. However, more importantly, this study has shown for the first time, that peptide vaccinated koalas are capable of producing a mucosal IgA immune response to whole *C*. *pecorum* EBs. This ability to identify the *C*. *pecorum* infectious agent is paramount to the successful development of an effective peptide-based anti-chlamydial vaccine. This demonstrates the specificity of our peptides to the key antigenic regions, required to stimulate a mucosal IgA response. Further showing that, despite any conformational 3D structures, our predicted epitopes of the target antigen molecule are surface exposed.

What was a surprising finding in our study, is that some of the peptide-vaccinated koalas had a stronger mucosal IgA antibody response to, rMOMP (G), peptides (P1 and P2) and whole chlamydial EBs, compared to MOMP-protein vaccinated koalas. One possible explanation for this could be the intracellular mechanisms by which antigens are processed within APCs, such as DCs. Synthetic peptides have been shown to follow a different intracellular pathway than proteins within DCs, with studies showing that DCs are more efficient at processing and directing synthetic peptides to MHC pathways, compared to whole proteins [23, 44, 45]. Furthermore, synthetic peptides have been shown to induce both CD4^+^ and CD8^+^ T cell immune responses in contrast to proteins, which have been shown to primarily induce CD4^+^ T cells and at lower levels [23, 45]. This further implies that synthetic peptides are more efficient at MHC class l cross-presentation, as outlined by Menager *et al*. (2014) [44].

To optimise immune responses and maximise DC activation and maturation, the addition of a Toll-like receptor (TLR) agonist is recommended for peptide based vaccines. Invading pathogens recognised by TLRs located on DCs trigger a response when bound, initiating DC maturation and immune responses [24, 46, 47]. To address this, we have combined our peptide vaccine with a Tri-Adjuvant containing Poly I:C, a known TLR agonist. Previously, it has been described that Poly I:C enhances immune responses, increases antigen uptake by DCs, [48, 49], and shows significant humoral and cellular immune responses [25]. Furthermore, a vaccine trial comparing adjuvants containing two different TLR agonists (Poly I:C and CpG), resulted in higher levels of IgA within the lungs of mice vaccinated with the Poly I:C adjuvant, and subsequently showed long lasting immunity [50]. This would suggest that poly I:C, combined with our peptide based vaccine, has contributed significantly to mucosal IgA antibody responses.

It has also been suggested that long lasting immunity could be attributed to the Tri-Adjuvant. Mice vaccinated with a live virus stimulated only short-lived immunity compared to mice vaccinated with a fusion protein of the virus combined with the Tri-Adjuvant, which demonstrated long term immunity [51]. They further showed that while both vaccinated groups of mice had similar responses to sera IgG, the fusion protein plus Tri-Adjuvant vaccinated group had a significantly higher IgA response than the live vaccinated group, in both the lung and lymph node [51]. The second component of the Tri-Adjuvant is a host defence synthetic peptide, innate defence regulator 1002 (IDR-1002), known for its ability to recruit immune cells, stimulate immature DCs, have antimicrobial activity, and anti-inflammatory properties [52, 53]. A recent study conducted to evaluate the ability of IDR-1002 demonstrated, both in-vitro and in-vivo, its ability to supress pro-inflammatory cytokines (tumor necrosis factor alpha and interleukin-6), and further showed a reduction in airway inflammation in IDR-1002 treated mice [54]. The third component of the Tri-Adjuvant is polyphosphazene, known for having immunostimulatory properties [55] and the ability to form non-covalent complexes with antigens enhancing uptake by APCs [56, 57]. Selecting an appropriate adjuvant to enhance the delivery and uptake of a peptide vaccine is crucial for optimal outcome. Here we have described the successful combination of a peptide vaccine antigen with a Tri-Adjuvant, resulting in strong mucosal IgA responses, necessary to combat a chlamydial infection.

Developing a peptide based vaccine is complex, with some challenges to consider. Although there are no commercially available peptide based vaccines as yet, there has been considerable progress towards this goal, for both the prophylactic and therapeutic effects [58, 59], particularly for the treatment of cancer [30, 60]. Progress towards the development of an anti-*Chlamydia trachomatis* peptide based vaccine have also been promising with results revealing strong humoral [42, 61] and cell mediated immune responses [61]. In considering future development, it has been suggested that the conjugation of both the antigen and adjuvant would ensure they are both delivered to the same APC, for optimal stimulation and maturation. Furthermore, having a better understanding about the functionality of the targeted epitope would also help in developing a more directed outcome specific to the required response.

In conclusion, we report for the first time, the success of a koala vaccine consisting of two synthetic peptides, derived from *C*. *pecorum* MOMP, in place of a previously used rMOMP protein based vaccine. We have shown that MOMP-peptide vaccinated koalas produced *Chlamydia*-specific antibodies that recognised not only rMOMP but importantly, also whole chlamydial EBs. We have shown that MOMP-peptide vaccinated koalas produced a *Chlamydia*-specific IgG and IgA immune response still seen at 26 weeks post-vaccination. Most importantly, this study has shown that MOMP-peptide vaccinated koalas can produce a stronger mucosal IgA response to whole EBs than MOMP-protein vaccinated koalas. Together, these results are promising and show the potential for the future development of a peptide based anti-*C*. *pecorum* vaccine that can be mass produced with precision for broader applications.
